# Determinants of delinquency in the Peruvian banking and microfinance system, 2015–2020

**DOI:** 10.3389/fsoc.2022.934724

**Published:** 2022-11-21

**Authors:** Julio Cesar Quispe Mamani, Miriam Serezade Hancco Gomez, Cristobal Rufino Yapuchura Saico, Juan Isidoro Gómez Palomino, Santotomas Licimaco Aguilar Pinto, Jorge Luis Vargas Espinoza, Fredy Toribio Chalco Vargas, Amira Carpio Maraza, Dominga Asunción Calcina Álvarez, Rolando Cáceres Quenta

**Affiliations:** ^1^Docentes de la Facultad de Ingeniería Económica, de la Universidad Nacional del Altiplano, Puno, Peru; ^2^Docente de la Facultad de Ciencias Contables, Escuela Profesional de Ciencias Contables, de la Universidad Nacional del Altiplano, Puno, Peru; ^3^Docente de la Facultad de Ciencias Sociales, de la Universidad Nacional del Altiplano, Puno, Peru; ^4^Docente de la Facultad de Ciencias Administrativas de la Universidad Andina Néstor Cáceres Velásquez, Juliaca, Peru; ^5^Docente de la Facultad de Gestión de Organizaciones de la Universidad Nacional Intercultural Fabiola Salazar Leguía De Bagua, Bagua, Peru; ^6^Docente de la Escuela Profesional de Medicina Veterinaria y Zootecnia de la Universidad Andina Néstor Cáceres Velásquez, Juliaca, Peru; ^7^Docente de la Facultad de Educación de la Universidad Nacional Amazónica de Madre de Dios, Puerto Maldonado, Peru; ^8^Docente de la Facultad de Ciencias de la Educación, de la Universidad Nacional del Altiplano, Puno, Peru

**Keywords:** financial management, bank, financial institutions, delinquency, financial resources

## Abstract

**Objective:**

The objective was to identify the variables that affect the delinquency rate in banking and microfinance institutions, between the periods 2015 and 2020, for which panel data models were used, considering the information registered in the banking and financial institutions to the level of Peru.

**Method:**

The methodological design used is quantitative, not experimental, with a descriptive-correlational design, applying the analysis of the data panel for each financial institution (Multiple Banking, Municipal Savings Banks), to observe the behavior over time for the same individuals.

**Results:**

It was determined that the behavior of the delinquency of microfinance institutions is having significant effects on the delinquency of loans, and macroeconomic variables like microeconomic variables do determine delinquency rates such as provisions, efficiency of analysts, financial income, liquidity in national currency, growth rate of Gross Domestic Product, and the level of unemployment, both for banks and for municipal savings and credit banks, explaining the study variables in 84.30% in the banking system and in 48.95% in the financial system with respect to delinquency.

**Conclusions:**

Macroeconomic and microeconomic variables are determining factors for the level of delinquency in financial institutions.

## Introduction

The financial system and the banking system play a decisive role in guaranteeing the economic growth of a country, and through the financial movements that are made in these, they provide to guarantee greater liquidity to families, companies, and the State. The process of developing the activities of financial and banking institutions contributes to the full development of the different activities that are planned mainly by the business sector, thus contributing to the growth of investment, economic growth, and the development of this, since the development of the economy in a decelerated way discourages the financial and banking system, and the non-existence of security in the system restricts toward the fluidity of the credits, on the contrary, if a greater fluidity occurs, a risk is generated that can affect the deterioration in its performance and development (Nikolaidou and Vogiazas, 2014; Uquillas and Flores, 2020; Uquillas and Tonato, 2022).

That is why financial institutions and banks play the role of stimulating the growth of the economy, which is complemented by the role played by central banks in the world since they formulate and implement the corresponding measures to guarantee administrative efficiency in the ratio of credit delinquency. The aforementioned shows that it is necessary to identify the determinants of delinquency in both systems, since the institution is responsible for guaranteeing the development of an optimal regulation in the loan portfolio and the behavior of macroeconomic variables will allow the implementation of necessary measures to prevent the deterioration of the delinquent portfolio, an issue that could occur in the event of the existence of an economic crisis or instability in the economic system (Lozano-Espitia et al., 2017; Ma and Diao, 2017; Valencia et al., 2017; Wadud et al., 2020).

When analyzing at the international level, the financial sector plays a vital role in a country's economy, and its lack can negatively affect the performance of companies; however, the financial sector does not always function properly, especially when there is a high level of information asymmetry, which causes damage to the economy (Benhayoun et al., 2013; Clavellina Miller, 2013; Almeida and Divino, 2015; Uquillas and Flores, 2020; Roslan et al., 2021). International information exchange offices have been introduced to reduce concerns about moral hazard and adverse selection issues in financial markets (Been et al., 2013; Acosta, 2017; Uquillas and González, 2017; Ghosh, 2019). Information sharing could mitigate problems related to foreign currency lending from companies in developing countries (Alessi and Detken, 2018; Kanungo and Gupta, 2021; Martens et al., 2021), as the financial strength of companies is set out in more detail to facilitate credit assessment (Aguilar et al., 2004; Rivas and Burgos, 2016; Ghosh, 2019).

The exchange of information through private agencies and public credit registries has become an important part of the development of the banking system globally, as they demonstrate that information exchange institutions help to increase the size of the credit market, which is complemented by the phenomena of the existence of the behavior of macroeconomic variables and industry-specific factors and exogenous factors to the same (Thirlwall, 1994; Barnett et al., 2006; Lizarzaburu et al., 2012; Bernad et al., 2013; Nikolaidou and Vogiazas, 2014; Climent Serrano, 2016).

However, in recent times, Peru has also paid attention to the challenges facing the banking and microfinance sector (Lizarzaburu Bolaños and del Brío, 2016). One aspect of this challenge has been to analyze the factors that affect the delinquency of this sector (De Graeve et al., 2008; Imai et al., 2010; Creel et al., 2015; Pradenas Soto and Vásquez Laurie, 2016). It is well-known that the economy of Peru had a fairly stable behavior compared to other countries since it showed economic and financial strength that allowed it to face many unstable scenarios that occurred in the world, and an adequate and efficient macroeconomic administration of the Central Reserve Bank allowed it to have adequate and optimal results, which translates into a solid and very solvent financial and banking system, despite having a deteriorated NPL ratio due to fluctuations in economic performance and the exchange rate (Espino and Carrera, 2006; Cermeño et al., 2011; Suárez, 2013; Lizarzaburu Bolaños and del Brío, 2016). According to the International Monetary Fund (IMF), non-performing loans are an impediment to economic activity, especially in countries where banks and microfinance companies play a very important role in financial intermediation (Tanna et al., 2011; Jiang, 2013; Piffaut and Rey Miró, 2016). In addition, the increase in non-performing loans reduces profitability, immobilizes bank capital, and increases financing costs (Quevedo, 2009; Clardy, 2012). A crucial challenge in policymaking is to address the threat of non-performing loans to unlock the supply of credit and ultimately promote economic growth (Murrugarra and Ebentreich, 1999; Loayza, 2019).

**Figure 1:**
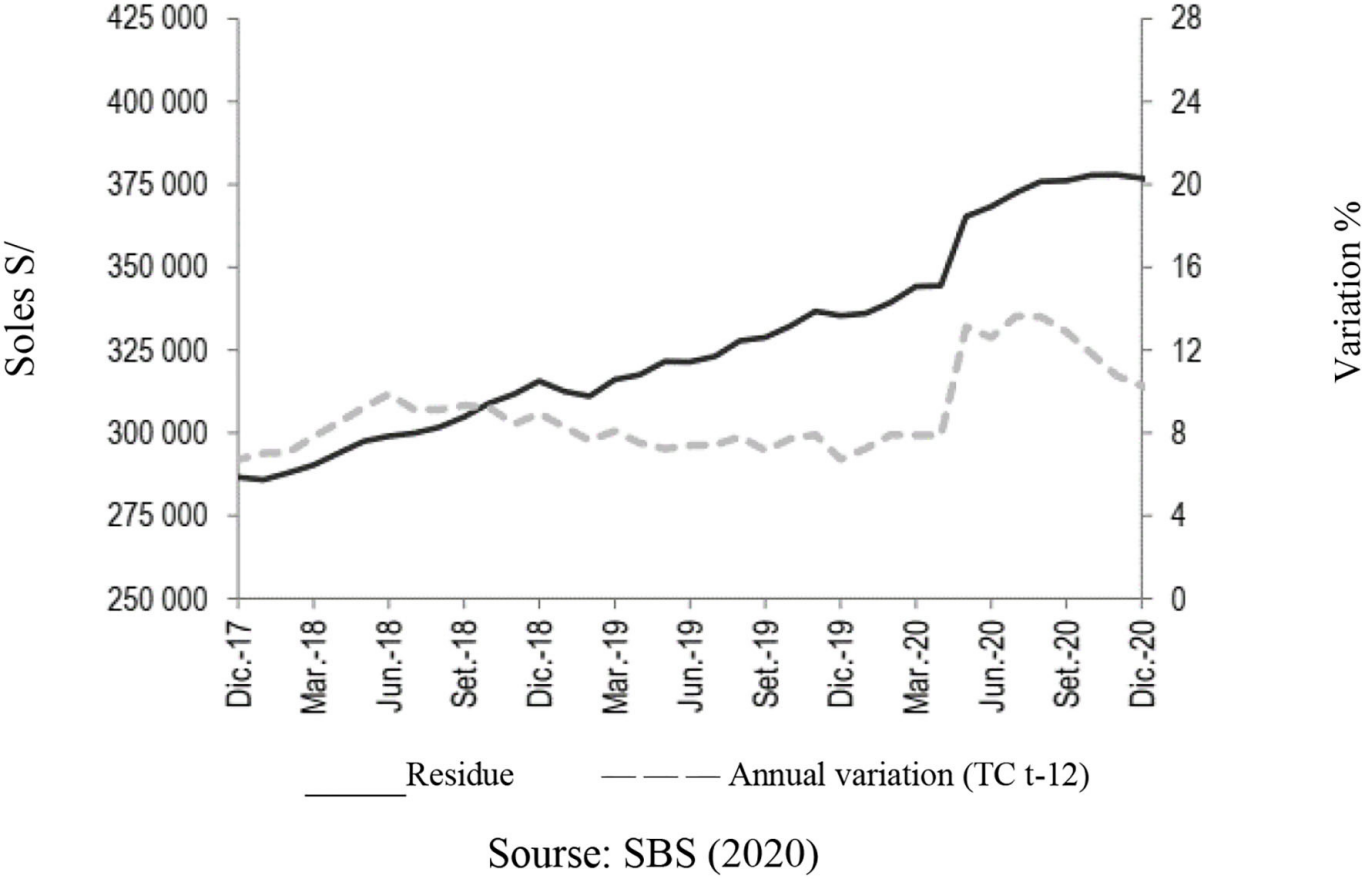
Financial system: direct credits. Source: SBS (2020).

According to the Superintendence of Banking and Insurance (SBS), the evolution of the financial system, as of December 2020, was made up of 56 companies with assets close to S/617 billion (equivalent to US$ 170 billion) (SBS, 2020; Cuba et al., 2021). About 83.6% of the assets are explained by multiple banks with a balance close to S/516 billion; also financial institutions, municipal savings banks, rural savings banks, and Edpymes represent 9.1% of total assets. Multiple banks account for the majority of loans and deposits in the financial system (86.5 and 81.7%, respectively) (Lahura, 2017; Arbulu, 2020). As for direct loans, the balance as of December 2020 reached S/376,901 million, which is higher by S/41,580 million registered in the previous 12 months, which represents an increase of 12.4% (Figure 1).

**Figure 2:**
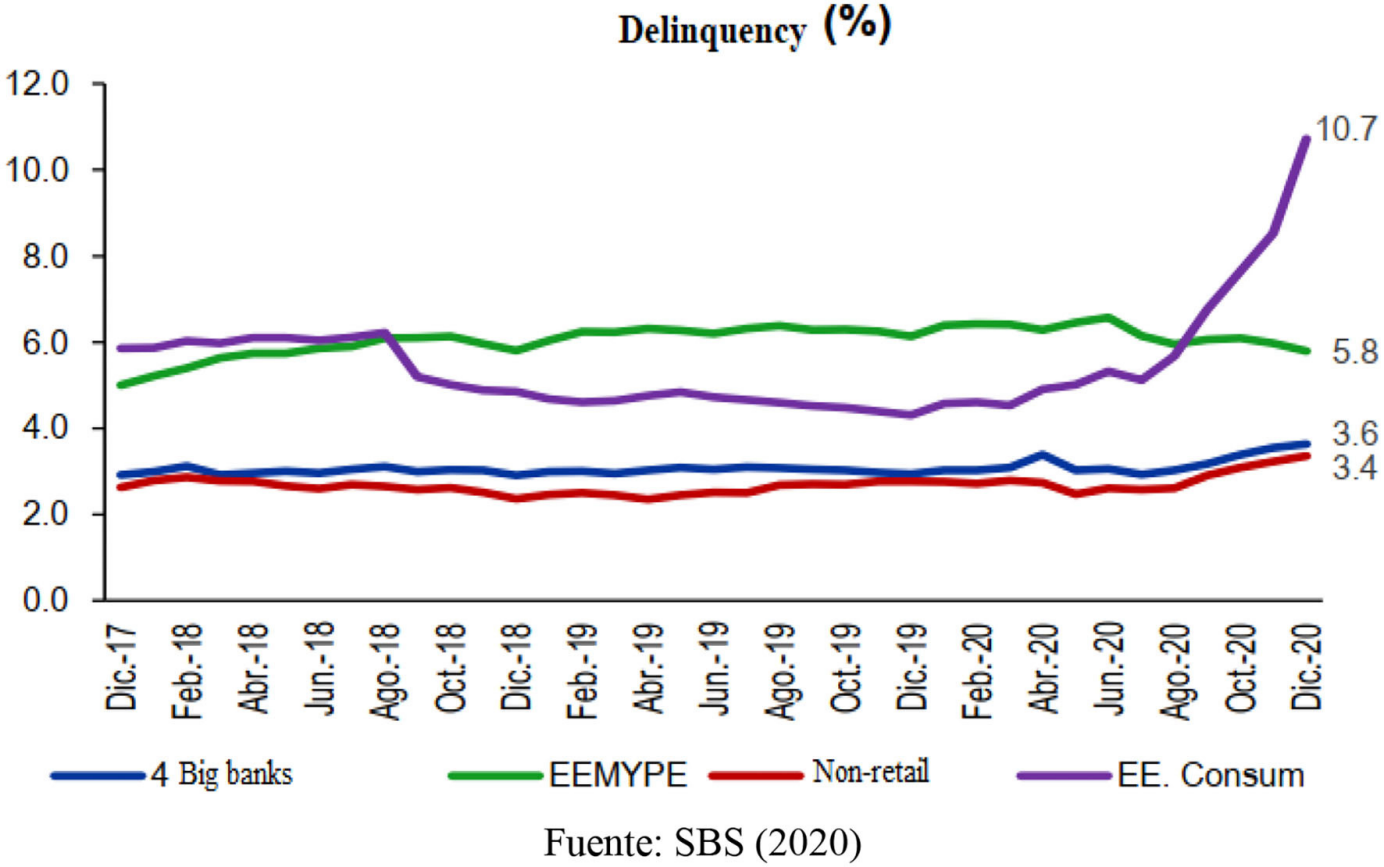
Delinquency behavior. Fuente: SBS (2020).

In addition, the delinquency required under the SBS method stood at 4.2% for all the companies with multiple operations at the end of December 2020. Similarly, the U.S. Consumer Department recorded the highest delinquency rate (10.7%), explained by the increase in the order book. It is followed by the US MSEs with delinquency of 5.8%, which showed a reduction in delinquencies compared to the previous year, mainly explained by the increase in placements throughout the last half of 2020 (Arbulu, 2020) (Figure 2).

Delinquency is shown as a serious drawback in the financial system that leads to collapse if it reaches outstanding levels and credit risk helps mitigate some information anomalies involved in credit; therefore, they provide the flow of credit to be more comfortable with respect to bad loans. It is very dangerous to have a high backlog that compromises its long-term viability and also endangers the system itself. As a result, this problem leads to liquidity problems that would complicate solvency and lead to the collapse of financial institutions (Been et al., 2013; Ghosh, 2019).

In addition, when a portfolio reclassifies the client to a much higher category, it represents a deterioration of its quality and at the same time endangers it which requires high level of provisions from the entities, and taking into account that of the total credit delivered, it supposes a fixed percentage as a basis to assume the debt when defaults occur. From the criteria established by the SBS, these are more favorable because it minimizes the risk of liquidity and solvency problems for entities, however, on the part of financial entities it harms since it affects the amounts of money generated (Arbulu, 2020).

Therefore, this research allowed us to answer the following questions: What are the determinants of delinquency in the Peruvian banking and microfinance system during the period 2015–2020? What are the macroeconomic variables that affected delinquency in the Peruvian banking and microfinance system? and What are the microeconomic variables that affected delinquency in the Peruvian banking and microfinance system?

The objectives of the research are to identify which are the variables that affect the delinquency rate in Banking and Microfinance Institutions 2015–2020, to determine which are the macroeconomic variables that influence the delinquency rate of banking and microfinance institutions, and to determine which are the microeconomic variables that influence the delinquency rate of banking and microfinance institutions.

In the same sense, the research hypothesizes that both microeconomic and macroeconomic variables do influence the level of delinquency in loans from banking and microfinance institutions, January 2015–December 2020; the most important microeconomic factors that influence the level of delinquency in Peruvian Banking and Microfinance System, January 2015–December 2020, are liquidity, leverage, and interest; and the most important macroeconomic factors that influence the level of delinquency in the Peruvian Banking and Microfinance System, January 2015–December 2020, are consumer price index, annual PIB variable, and unemployment rate.

## Literature review

According to Gonzales M. I. C. (2012), in an economy, there is an interaction of confidence between delinquency and cheap cycles, finding evidence that for the recessionary phases of the economy, delinquency grows, and this explains why in the recession stage of an economy, companies and families face critical and chaotic financial occasions. The opposite occurs when there are expansionary phases where an area of the capital increase is shown, so the development of credit is accelerated; therefore, the income to all types of credits increases resulting in a reduction in the rigor of credit income. While Arana and López (2019) established that the more the delinquency rate increases in the agency, more provisions increase proportionally causing the profitability of the institution to be reduced. In this way, the researcher makes it clear that there are no good guarantees of causes of delinquency.

For Aguilar and Camargo (2004), macroeconomic variables and microeconomic variables do determine delinquency in the banking system in Peru, complementary to the above, highlighting that the internal variables of the banking system also have repercussions on delinquency; they found that the country's economic cycle has an inverse relationship with delinquency since an increase in economic growth has the effect of decreasing the delinquency of the banking system, but if the country's economy is in a process of recession, then that delinquency ratio will have a tendency toward increase. In addition, the debt variable is not relevant in delinquency, since the share of credit in economic growth is not significant with respect to other countries.

On the other hand, Murrugarra and Ebentreich (1999) consider that the determining variables of delinquency in Edpymes are the credits per person and the delinquency of the department. Likewise, the importance of the management of credits per person is relevant so it would indicate that the management of credits from the beginning of the operations of the Edpymes is important.

According to Cermeño et al. (2011), the interest rate of loans, liquidity, and the intermediation of funds that is measured through the credit-to-deposit ratio have a direct effect on the delinquency rate. This is because the economic development of the country measured through the percentage variation of economic growth affects inversely the delinquency of the financial system (Municipal Savings and Credit Banks).

Rodríguez (2015) indicates that one of the possible explanations for the credit restriction maintained by the bank is that, given the current systems of identification and rating of credit applications, the delinquency rate of borrowers becomes a variable that negatively and significantly impacts the profit obtained by banks.

According to Sellan (2019), delinquencies have a positive relationship with the level of indebtedness of economic agents and the real exchange rate. It also showed that the economic cycle has positive effects on the delinquency rate, and in the case of the liquidity restriction measured by the active interest rate, the depreciation rate of the national currency and the increase in indebtedness that companies have important effects on the delinquency rate.

Loayza (2019) examined the determinants of the fulfillment of its credits for a sample of small and medium-sized enterprises. All the changing ones that reflect the fulfillment of the company with its financial credits point out that the small and medium-sized enterprises that must comply with their financial obligations within the predetermined period are those that obtained credits with lower interest rates, they have higher returns in their occupations, higher trading activity and liquidity, and also have a longer life. The review work also provides empirical proof in the post of the theories that justify microcredit, which is beyond having a different orientation to that of commercial bank credit.

## Data and methods

### Design, approach, and research variables

The design of this research was descriptive-correlational type of non-experimental research, with a quantitative approach, since the study variables were not manipulated (Sampieri and Collado, 2010; Mendoza Bellido, 2014).

### Technique and instruments used

For the present research, the econometric model of data panel with random effects was applied, since a sample of interest (banking and financial entities) was included between the period 2015 and 2020, making a combination of two types of data (temporal and structural dimension), for which the statistical program Eviews 14.0 was used (Drukker et al., 2011).

### Analysis and sample period

The period of analysis in the present research was from 2015 to 2020, where a total of 37 banks, municipal savings banks, and financial institutions were studied, considering a margin of error of 5% and a confidence level of 95%. The sources of data in this information were the official institutions of the Superintendency of Banking and Insurance (SBS), the Central Reserve Bank of Peru (BCRP), and the National Institute of Statistics and Informatics (INEI).

### Procedure

At first, the independent variables under study were described and then a correlation analysis was made with the dependent variable (Sampieri and Collado, 2010). In the present research, a data panel analysis was performed for each financial institution (Multiple Banking, Municipal Savings Banks) to observe the behavior over time for the same individuals, and the model is formed as follows:


$$\begin{array}{l} Delinquency_{it}=\beta_{1}+\beta_{2}apalanc_{it}+\beta_{3}CD\;work_{it}+\beta_{4}ROA_{it}\\ \;+\;\beta_{5}Ingf_{it}+\beta_{6}Liqmn_{it}+\beta_{7}TAMN_{it}\\ \;+\;\beta_{8}IPC_{it}+\beta_{9}VARPIB_{it}+\beta_{10}Peadesoc_{it}\\ \;+\;e_{it} \end{array}$$

Where, for the establishment of the variables, the following were applied:

Leverage is equal to contingent assets and risk-weighted credits/Capital, provision coverage is equal to the level of provisions/Back portfolio, direct returns by number of workers (CD_work), economic profitability (ROA), net financial income (Ingf) is equal to financial income /Total income, the liquidity ratio in national currency (Liqmn), the weighted average active interest rate (Tamn), inflation, as measured by the consumer price index (CPI), Gross Domestic Product Growth Rate (VarPIB), and level of unemployment measured by the economically active population (EAP) unemployed (Peadesoc).

## Results

### Analysis of delinquency variables in the Peruvian banking and microfinance system

**Figure 3:**
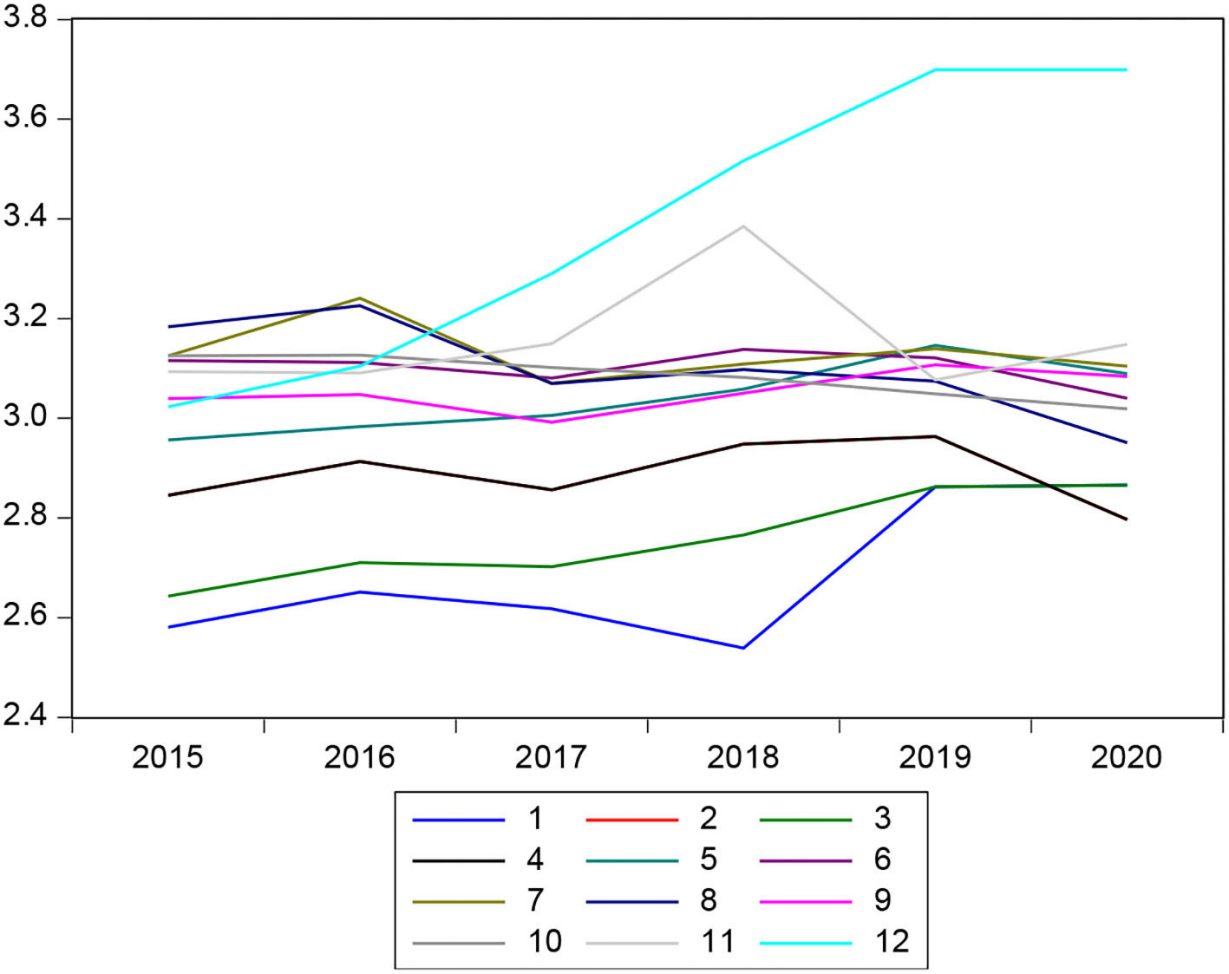
Behavior of the delinquency rate.

The delinquency in multiple banks in Peru in recent years had a tendency to increase year after year, due to different external and internal factors, and as can be seen in Figure 3, the behavior of the delinquency rate between 2015 and 2020 was oscillating between 2.5 and 3.07% on average. In the case of banks, in recent years, there was excess liquidity and it is for this reason that delinquencies in some institutions have been increasing proportionally. We denote that microeconomic variables are important because not all financial institutions share the same variables, so in the figure, it is observed that entity number 12 has the highest level of delinquency at the end of 2020.

**Figure 4:**
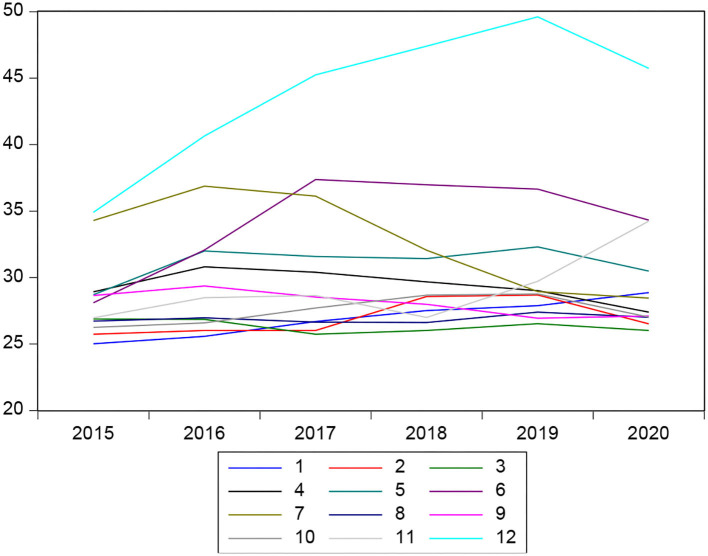
Liquidity behavior of financial institutions.

Regarding financial liquidity, it can also be observed that in recent years, it has an upward trend in the case of some financial institutions, and the results of the estimate shows us that this variable is significant with the variable dependent on delinquency since if there is a direct relationship, while a financial institution has high levels of liquids, delinquency levels also tended to increase. In addition, the hypothesis raised from the research that affirms that microeconomic variables do determine the level of delinquency is verified (Figure 4).

**Figure 5:**
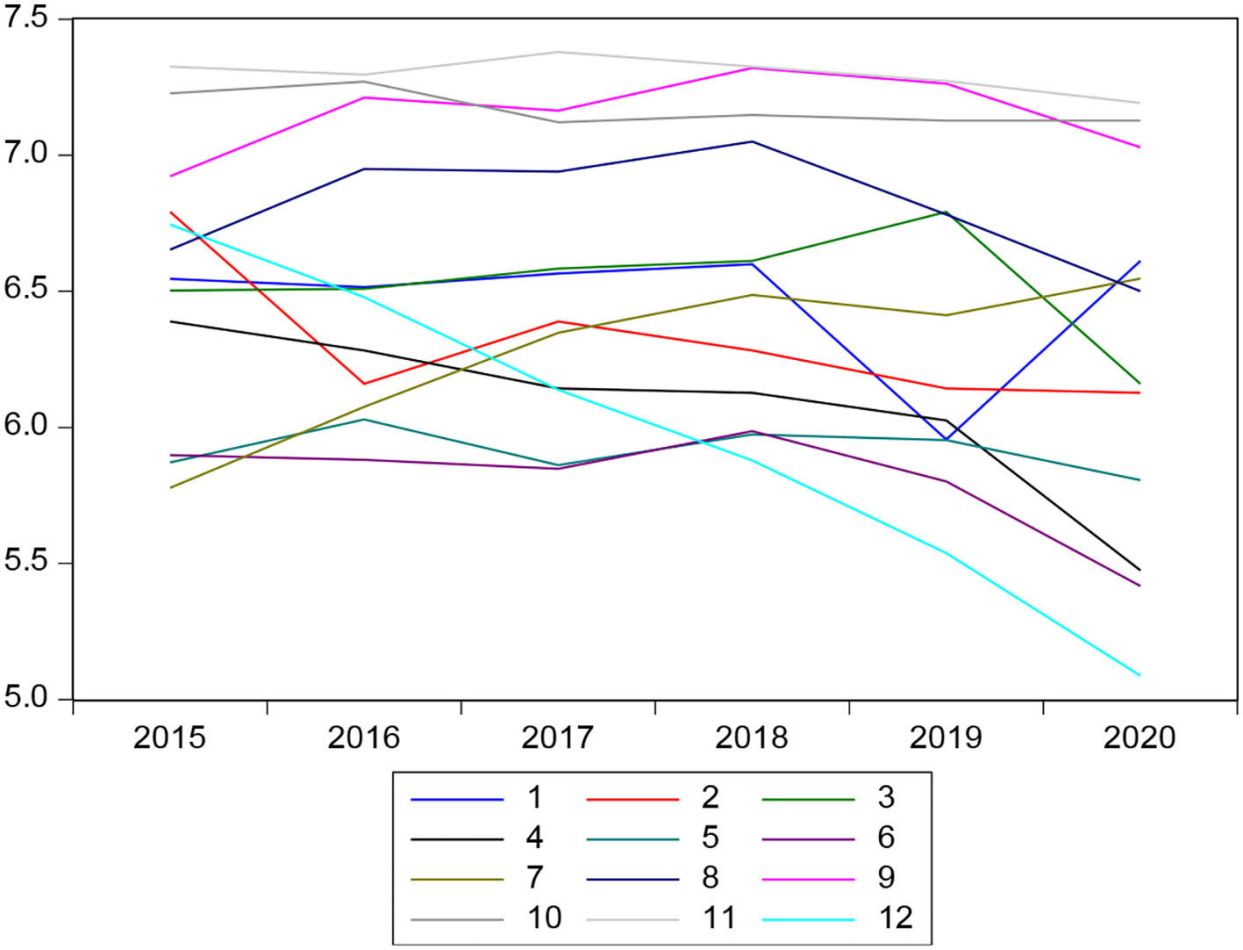
Crime behavior in the CMAC.

The delinquency in the Municipal Savings and Credit Fund (CMAC) as observed in recent years had a different behavior with respect to banks, its trend is ambiguous for each entity. In this sense, it can be observed that the behavior of the delinquency rate is different from that of banks with an average of 5.1 and 7.04%, as the delinquency rate is higher in these financial institutions. These do not follow a pattern and it is shown that there is a higher delinquency rate because the CMAC has greater risks when making loans because they ask for fewer guarantees than the common bank (Figure 5).

**Figure 6:**
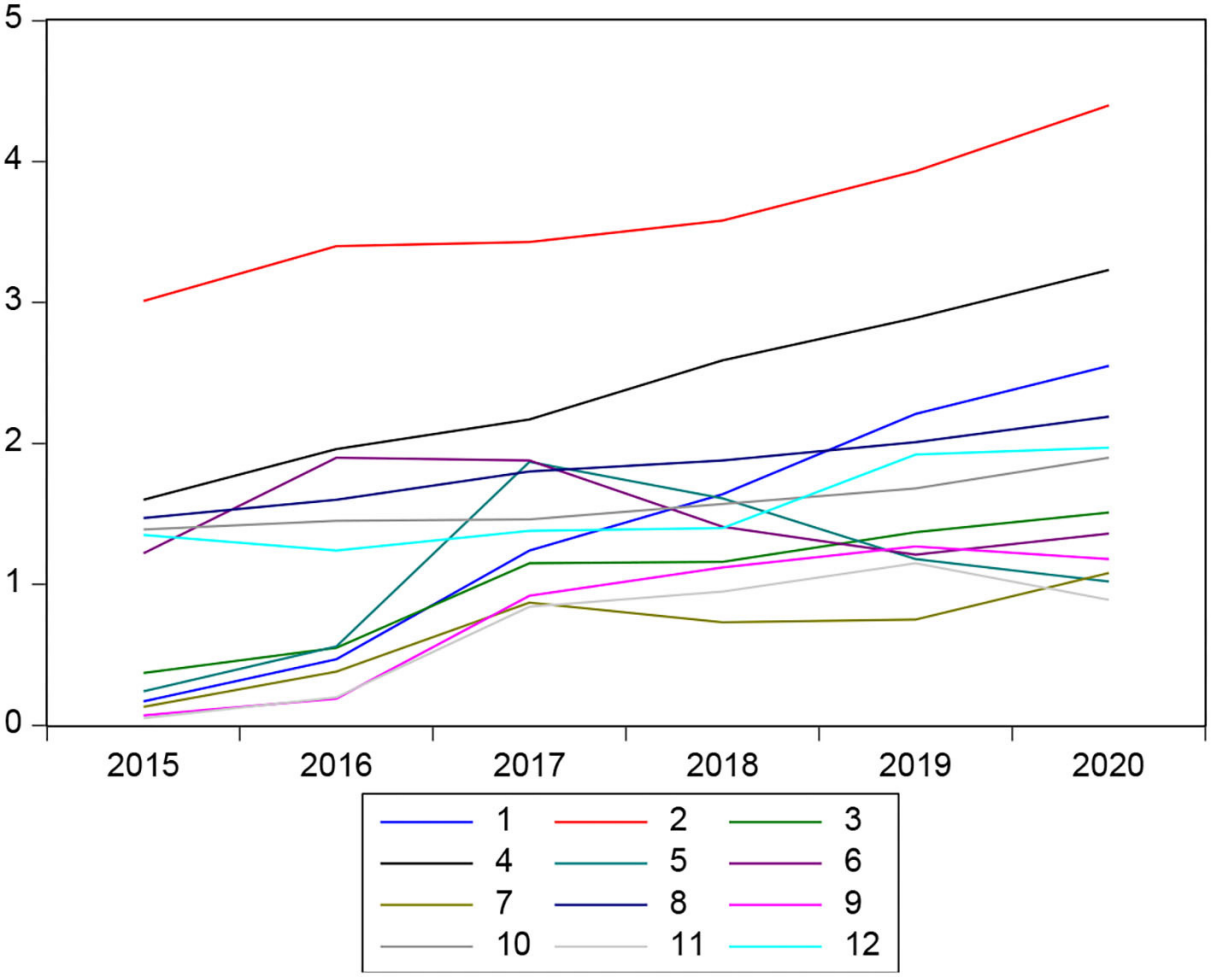
Change in gross domestic product (PIB).

Regarding macroeconomic variables, the hypothesis raised in the research is also verified, which states that macroeconomic variables such as gross domestic product and the consumer price index, among others, do determine the level of delinquency because the higher the levels of gross domestic product, the lower the delinquency rates; this is because people have better ability to pay (Figure 6).

### Econometric analysis for the determinants of the level of delinquency in banking and microfinance institutions of the financial system

The present research performed two econometric models one for banks and the other for the boxes and when making the estimates, it was concluded that the estimates of both models with data panel were better suited to the random effects model presented below.

In the case of banks (Table 1):

**Table 1:** Determinants of delinquency in banks.

**Dependent variable: Delinquency**				
**Method: Panel data – random effects**				
**Variable**	**Coefficient**	**Standard error**	***T*-statistic**	**Prob**.
Constant	3.305691	0.277375	11.91777	0.0000
Risk-weighted contingent assets and credits/Equity (Leverage)	−0.044995	0.03739	−1.203414	0.2333
Provisions/Backlog (Provisions Coverage)	−0.014366	0.001273	−11.28191	0.0000
Direct credits by number of workers (CD work)	0.000312	2.65E-05	11.76509	0.0000
Economic profitability (Roa)	0.001293	0.000764	1.691632	0.0957
Liquidity in national currency (Liqmn)	0.026902	0.002544	10.57362	0.0000
Growth rate of Gross Domestic Product (VarPIB)	0.068629	0.012668	5.417588	0.0000
Level of unemployment (Peadesoc)	0.025573	0.009569	2.672553	0.0096
Consumer price index (IPC)	−0.035904	0.033712	−1.065019	0.2909
**Effect specification**				
	**Tests**		**Standard deviation**	**RHO**
Random cross section			0	0
IR			0.069883	1
**Weighted statistics**				
*R*-square	0.860742		Mean dependent variable	3.022804
Adjusted R-square	0.843058		Standard deviation of dependent variable	0.212441
S.E. of regression	0.084161		Sum to square of residue	0.446229
*F*-statistic	48.67458		Durbin Watson Statistician	1.247308
Prob (F-Statistics)	0.000000			

Source: Own elaboration.

It is observed that most of the variables are significant and have a good level of joint significance, likewise almost most of them are relevant. In addition, it is demonstrated that the model is adequate and consistent with the expected signs and with variables with a high level of significance both individually and as a whole; these variables are provisions, number of workers, liquidity in national currency, variation in PIB, and the level of unemployment that explain the probability of the increase or decrease in delinquency.

The estimated leverage ratio was −0.044995, of which it can be determined that it is not significant individually because its probability 0.2333 > 0.05, determining that it indirectly influences crime; therefore, if we have an increase of one percentage unit in leverage, the delinquency will be reduced by −4.4995% to delinquency. On the other hand, it has been used as an indicator of provision coverage, so we can deduce that it is significant individually for the model, having a value less than its probability 0.0000 < 0.05, whose elasticity value is −0.014366, that is, for each percentage point that tends to decrease the coverage of provisions, delinquency tends to increase by 1.43%, of which it is observed that, if the quality of the portfolio deteriorates and the bank does not readjust the relevant provisions, the coverage ratio decreases. In addition, for the bank, delinquent customers are more expensive because of the obligation of legal provisions established by the supervisory body of the system. The derivative sign would be expected to be positive, as it would be higher provisions. In the case of direct credits by number of workers (CD-work), it was significant individually with a coefficient of 0.000312, that is, for each additional credit that an analyst attends, its delinquency increases by 0.0312%, which is intended to say that the greater the number of credits that an analyst attends, the higher the delinquency rate.

Economic profitability (ROA) is an indicator that measures the efficiency and management of financial companies. It follows that the higher the level of ROA, the lower the level of delinquency. The estimate provides a positive and not individually significant coefficient of 0.001293, with a probability value >0.05. Therefore, if we have an increase of one percentage unit in economic performance (ROA), the delinquency will increase by 0.1293. In the case of liquidity in national currency, it is significant with a probability <0.05, so having a coefficient of 0.026902, we can say that if a financial institution has an abundance of liquidity, it tends to lend more frequently; therefore, if we have an increase of one percentage unit of liquidity, then the delinquency increases by 2.69%.

Regarding the macroeconomic variables, the PBI growth rate (VarGDP) shows a positive impact, with a coefficient of 0.068629, being this individually significant variable has a value less than its probability 0.0000 < 0.05, determining that the growth rate of the PBI directly influences delinquency; therefore, if we have an increase of 1%, then we will have an increase in delinquency of 6.86%. This means that an improvement in the level of economic activity reflects an improvement in the quality of the portfolio, and in the face of unfavorable economic fluctuations, agents will have difficulty amortizing their credits.

The variable level of unemployment is considered important because it incorporates labor market conditions by disclosing employment indices that reflect changes in agents' liquidity constraints. This variable is statistically significant and has a probability value of <0.05; so its estimated coefficient was 0.025573, of which we empirically verified that high levels of unemployment increase the delinquency rate. That is, for every thousand Peruvians who enter the economically inactive population, the delinquency rate tends to increase by 2.55%. In addition, the consumer price index (CPI), as a variable part of the general model, was not significant, given that the value of the probability of its coefficient is >0.05, with an elasticity of −0.035904. Determining in this way that inflation affects the purchasing power of people who, in the face of an inflationary crisis, therefore, cover their basic needs first, such as food and not debts.

In the case of the Municipal Savings and Credit Banks (CMAC), the results obtained are as follows (Table 2):

**Table 2:** Determinants of delinquency in financial institutions.

**Dependent variable: Delinquency**				
**Method: Panel data – random effects**				
**Variable**	**Coefficient**	**Standard error**	***T*-statistic**	**Prob**.
Constant	20.77118	3.726071	5.574553	0.00000
Provisions/Backlog (Provisions Coverage)	−0.01563	0.004804	−0.253516	0.0018
Direct credits by number of workers (CD_Work)	−0.004834	0.000657	−7.361485	0.0000
Financial Income/Total Income (financial income)	−0.094393	0.041249	−2.288386	0.0254
Liquidity in national currency (Liqmn)	0.096229	0.01667	5.772404	0.00000
Consumer Price Index (IPC)	0.161128	0.082917	1.943254	0.00000
Growth rate of Gross Domestic Product (VarPIB)	−0.189095	0.037136	−5.091973	0.00000
Average National Currency Interest Rate of_BCR	−0.132298	0.080518	−1.64308	0.1053
**Effect specification**				
	**Tests**		**Standard deviation**	**RHO**
Random cross section			0.139056	0.3941
IR			0.172411	0.6059
**Weighted statistics**				
*R*-square	0.539897		Mean dependent variable	2.927509
Adjusted *R*-square	0.489573		Standard deviation of dependent variable	0.333495
S.E. of regression	0.238262		Sum to square of residue	3.633213
*F*-Statistic	10.72848		Durbin Watson Statistician	1.413523
Prob (*F*-statistic)	0.000000			

Source: Own elaboration.

The coverage of provisions in the financial system is significant and has an inverse relationship with delinquency, since its probability is <0.05 (0.0018 < 0.05). It has a coefficient equal to −0.015630, that is, for each percentage point that tends to decrease the coverage of provisions, delinquency tends to increase by 1.56%; hence, it is observed that, if the quality of the portfolio deteriorates and the bank does not readjust the relevant provisions, the NPL ratio decreases. The bank costs more delinquent customers because of the obligation of legal provisions established by the supervisory body of the system. The derivative sign would be expected to be positive, since the greater the provision, there will be decrease in the level of delinquency. The variable direct credits by number of workers (CD-labor) was also significant and the coefficient obtained was 0.0048, that is, for each additional credit that an analyst attends, its delinquency increases by 0.48%, which shows that the greater the number of credits that an analyst attends, the higher the delinquency rate (Table 2).

The variable financial income shows the benefits of financial intermediation, so in the present research, it is significant and shows a coefficient of −0. 094393, demonstrating an inverse relationship with delinquency and it can be determined that if it increases by 1%, then the delinquency rate will decrease by 9.43%. Considering that liquidity in national currency is also significant with a probability <0.05 and with a coefficient of 0.096229, we can deduce that if a financial institution has an abundance of liquidity, it tends to lend more frequently; therefore, if we have a 1% increase in the liquidities in national currency, then the delinquency will increase by 9.62%. In the case of the consumer price index (CPI), this is significant with a probability <0.05, where the coefficient amounts to 0.161128. Therefore, for an increase of this coefficient by 1%, the delinquency will increase by 16.11%, having a very outstanding effect on the proposed model (Table 2).

As for the macro variables, the PIB growth rate shows a negative impact with an elasticity of −0.189095. This variable is significant individually having a value less than its probability 0.0000 < 0.05, determining that if the variable inversely influences delinquency so we have a decrease for each percentage point in PIB, then we will have an increase in delinquency of −18.90% (Table 2).

Therefore, as in the estimates for banks, the macroeconomic variables that were not significant are the interest rate in national currency. Unlike banks, the growth rate of gross domestic product had a coefficient of −0.1890 and exceeds the elasticity derived in the case of banks, thus showing that an improvement in the level of economic activity reflects an improvement in the quality of the portfolio (Table 2).

## Discussions

As can be seen in the comparison of the variables, these do not affect equally in the case of banks and municipal savings banks, and there are undoubtedly differences, even if they are minimal, but they exist. In addition, there are other variables considered, but these do not explain the determinants of the delinquency rate and, therefore, were discarded from the efficient model. The vast majority of the significant variables are microeconomic variables, which indicates that the entities themselves are responsible for the quality of their portfolio and that this is also the reason why it deteriorates, unnecessarily creating risks that in the long run will only highlight the low monitoring when reviewing the credits that will be granted. In the case of macroeconomic variables, we observed that indicators such as the growth rate of the Gross Domestic Product and the level of unemployment have a similar influence on the determinants of the delinquency rate in the Peruvian financial system.

After determining the econometric estimates, the main variables were discussed, starting with the variable direct credits by a number of workers (Cd_work) with a coefficient of 0.00031 in the banking system and −0.0048 in the financial system, thus demonstrating coincidences with the results obtained by Coral and Francis (2010) since in his research, he also finds this variable significant, thus explaining that the efficiency of the advisors does influence the delinquency rate for both banks and municipal savings and credit banks.

Regarding the variable economic profitability (Roa), in the research of Coral and Francis (2010), it is not significant and has no relation to the variable dependent on delinquency. However, in the present investigation, the ROA variable turned out to be directly proportional to the variable dependent on delinquency which may be due to the fact that the return on assets on capital in past years was very low compared to the years studied to the present research, therefore, he does not agree with the author in the mention regarding that variable.

The variable liquidity in national currency in the research is statistically significant, both in the banking and financial system with a coefficient equal to 0.026 and 0.096, respectively, coming to coincide with the position research that mentions that the higher the liquids, the municipal agencies tend to place more credits with weak controls directly affecting the delinquency rate of these same entities (Saurina Salas, 1998). Another variable that is statistically significant is the level of unemployment but in the banking system, where the behavior of people is to stop paying their debts when they do not have a stable job directly affects the delinquency rate of banks that agree with Coral and Francis (2010) research who comes to the same conclusion.

Finally, the growth rate of the Gross Domestic Product shows that it is statistically significant in the research for both banks and municipal savings and credit banks, given that it has a directly proportional relationship with the delinquency rate in the banking system and an inversely proportional relationship with the delinquency rate in the financial system, not achieving the same results with Saurina Salas (1998), Coral and Francis (2010) deducing that for the purposes of the present years, the growth rate of the Gross Domestic Product does influence the rate of delinquency in the last 5 years either directly or indirectly.

## Conclusion

Macroeconomic variables like microeconomic variables do determine delinquency rates such as provisions, analyst efficiency, financial income, liquidity in national currency, growth rate of Gross Domestic Product, and the level of unemployment, both for banks and for municipal savings and credit banks, explaining the study variables in 84.30% in the banking system and 48.95% in the financial system with respect to delinquency. The macroeconomic variables that influence banks are the PBI growth rate and the level of unemployment, from which it can be deduced that these have an impact because the most unemployed people will have less liquidity and therefore less capacity to meet their debts, while that in the municipal savings, only the PBI growth rate influences, this is because the variations in the study increase or decrease that the production of an economy experiences in certain periods (years or quarters) reason for which yes or yes This variable affects both banks and municipal savings banks.

The microeconomic variables that determine the evolution of delinquency for banks are leverages because the debt mechanism is used to increase the amount of money people have as their debts, and therefore, the delinquency rate will have a tendency to decrease. The efficiency of the analysts also influences delinquency, because the more credits they are asked to place, the less guarantees they will ask for and therefore the delinquency rate will increase and finally the liquidity in national currency determines the delinquency rate because the more money you have one more banking agency will be sought by clients to be able to borrow without guarantees or other types of controls.

In the case of municipal savings banks, the provisions are significant since their liquids are adjusted compared to banks, so if municipal savings banks do not properly provision their loan portfolios, the delinquency rate tends to increase significantly. The variable liquidity in national currency is also significant for municipal savings banks similar to in banks and therefore it is concluded that for all variables, each entity is responsible for carrying them out efficiently and effectively and systematically managed to control in the best possible way the delinquency rates in financial institutions in Peru.

The limitation that the investigation had is the access to the detailed information of the banking and financial institutions since these restrict most of the information. The institutions where they publish financial and banking information, such as the Ministry of Economy and Finance, the Central Reserve Bank and the Superintendence of Banking, and Insurance and AFPs, consolidate the information, but it is restricted, which makes it difficult to carry out investigations in a timely and opportune manner. In addition, the existing information is not easily accessible to researchers, since the Superintendence of Banks, as a regulatory entity, regulates and supervises the information generated by financial and banking institutions.

Finally, when considering the limitation of access to information to carry out this type of research, it is suggested to continue conducting research including specific topics on the determinants of delinquency from the point of view of the client, since it would be necessary to know the other side of the coin. In addition, it is suggested to carry out research perspectives of clients and officials on the quality of service provided to the client, so that from them, strategies can be generated to reduce the level of delinquency existing in banks and microfinance institutions in Peru and the world. It is also necessary to carry out research on the credit risk that may exist in banking and financial institutions in post-pandemic times, given that the economic fragility of families and companies are still latent after the consequences suffered by COVID-19. In addition, it is suggested to investigate the moral hazard and adverse selection that may exist in the financial system and in the micro-financial system, since it could allow demonstrating the causes and consequences that can be generated and affect both the clients and the institutions involved.

## Data availability statement

The original contributions presented in the study are included in the article/supplementary material, further inquiries can be directed to the corresponding author/s.

## Author contributions

JQ conceived and carried out the study. MH and CY contributed as study mentors. JG, SA, JV, FC, AC, DC, and RC participated in the design, data analysis, and writing of the research paper. All authors reviewed and approved the research paper. All authors contributed to the article and approved the submitted version.

## Conflict of interest

The authors declare that the research was conducted in the absence of any commercial or financial relationships that could be construed as a potential conflict of interest.

## Publisher's note

All claims expressed in this article are solely those of the authors and do not necessarily represent those of their affiliated organizations, or those of the publisher, the editors and the reviewers. Any product that may be evaluated in this article, or claim that may be made by its manufacturer, is not guaranteed or endorsed by the publisher.
